# Dietary n-3 Polyunsaturated Fatty Acid Intakes Modify the Effect of Genetic Variation in *Fatty Acid Desaturase 1* on Coronary Artery Disease

**DOI:** 10.1371/journal.pone.0121255

**Published:** 2015-04-07

**Authors:** Fengqiong Liu, Zhongxia Li, Xiaofei Lv, Jing Ma

**Affiliations:** 1 Department of Epidemiology and health statistics, School of Public Health, Fujian Medical University, Fuzhou, Fujian, China; 2 Department of Nutrition, School of Public Health, Sun Yat-Sen University, Guangzhou, Guangdong, China; Max Delbrueck Center for Molecular Medicine, GERMANY

## Abstract

**Background:**

Previous studies suggested that dietary fatty acids could affect blood lipids by interacting with genetic variations in *fatty acid desaturase 1 (FADS1)*. However, little is known about their direct effects on coronary artery disease (CAD). The aim of this study was to evaluate whether dietary n-3 long-chain polyunsaturated fatty acids (LCPUFAs) -eicosapentaenoic acid (EPA) and docosahexaenoic acid (DHA) could modulate the effect of *FADS1* rs174547 polymorphism on CAD.

**Methods:**

*FADS1* single-nucleotide polymorphisms rs174547 genotypes were measured in 440 CAD patients and 838 healthy controls. Dietary EPA and DHA intakes were assessed with a validated quantitative frequency food questionnaire. The association between *FADS1* rs174547 and CAD was estimated using logistic regression under both dominant and additive genetic models. The interactions between rs174547 polymorphism and LCPUFAs were analyzed by using multiple logistic regression and the “genotype × n-3 LCPUFAs” interaction term was included into the model.

**Results:**

We found that the minor *T* allele of *FADS1* rs174547 increased CAD risk (OR = 1.36, 95%CIs 1.03-1.80), and observed significant interaction between rs174547 and dietary EPA intakes on CAD (*P*-interaction = 0.028). The *T*-allele was only associated with higher CAD risk among individuals with lower dietary EPA intakes, but not in those with higher EPA intakes. Similarly, significant interaction was also observed between rs174547 and dietary DHA intakes on CAD (*P*-interaction = 0.020).

**Conclusions:**

Dietary n-3 LCPUFA intakes could modulate the association between *FADS1* rs174547 polymorphism and CAD. High dietary n-3 LCPUFA intakes could negate the unfavorable effect of genetic variation in *FADS1* on CAD in middle-aged and elderly Chinese population.

## Introduction

**Fig 1:**
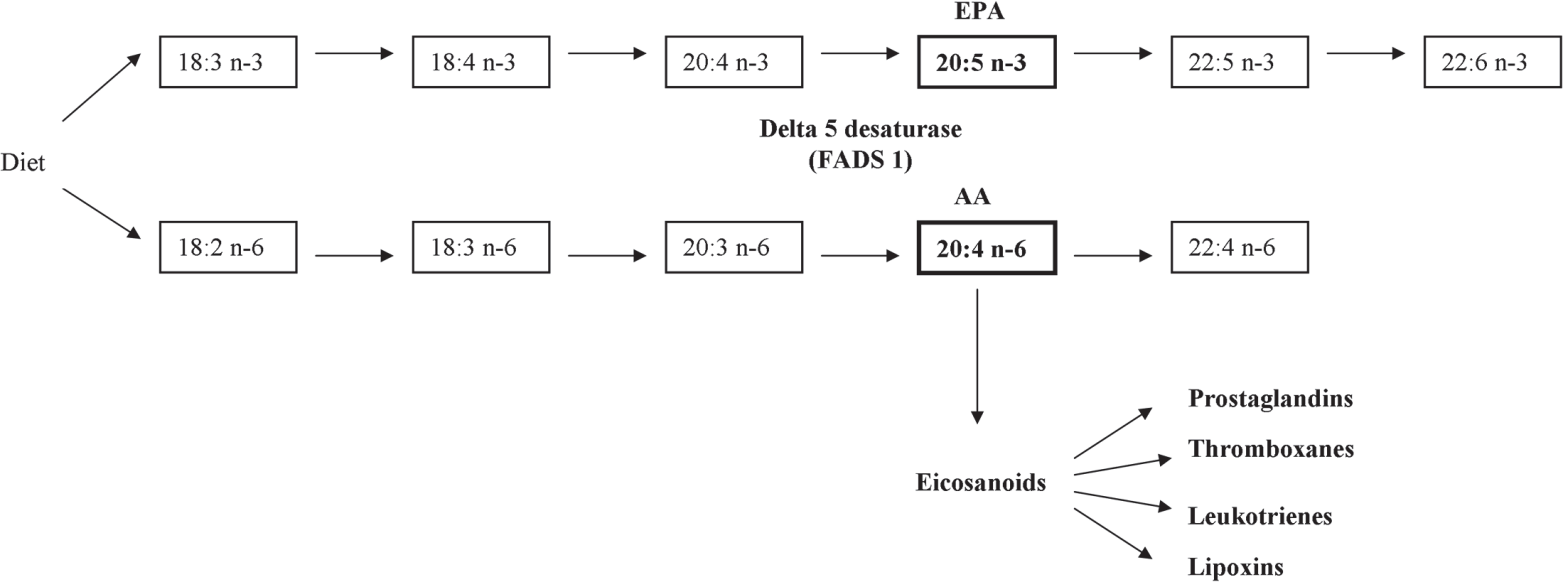
The n-3 and n-6 PUFAs metabolism pathways. AA, arachidonic acid; EPA, eicosapentaenoic acid; FADS: fatty acid desaturase.

Fatty acid desaturases (FADS) are a cluster of enzymes coded by *FADS* genes. The delta-5 desaturase is a rate-limiting enzyme involved in the endogenous metabolism of n-3 long-chain polyunsaturated fatty acids (LCPUFAs)— 20:4 n-6 (arachidonic acid, AA) and 20:5 n-3 (eicosapentaenoic acid, EPA) by introducing a double bond at the delta-5 position of 20-carbon fatty acids—20:3 n-6 and 20:4 n-3, respectively [1–2] **(Fig 1)**. It was reported that the minor allele of genetic single nucleotide polymorphism (SNP) rs174547 in *FADS1* gene was associated with increased triglyceride and decreased high density lipoprotein (HDL) cholesterol [3–4], as well as increased coronary artery disease (CAD) risk [5].

Dietary n-3 LCPUFAs have been well reported to have protective effects on the initiation and development of CAD [6–7]. American Heart Association has recommended to increase fish oil intake for the primary and secondary prevention of CAD [8], for the reason that fish oil is rich in n-3 LCPUFAs, especially in EPA and docosahexaenoic acid (DHA). The potential benefit effects of n-3 LCPUFAs on CAD have been testified in a number of epidemiologic studies [6, 9–10]. Hellstrand *et al*. [11] reported that the minor allele of rs174547 was associated with lower low density lipoprotein (LDL) cholesterol only among the subjects in the lowest tertile of n-3 polyunsaturated fatty acid (PUFA) intakes. However, most of the previous studies focused on conventional risk factors (i.e., blood lipids) of CAD [11–13], while little is known about the direct effect of n-3PUFA ×*FADS* genotype interaction on CAD. Therefore, the aim of the present study was to explore whether n-3 LCPUFA intakes (EPA and DHA) could modulate the association between SNP rs174547 in *FADS1* and CAD in the middle-aged and elderly Chinese population.

## Methods

### Study population

The design and methodology of this case-control study were described as previously [5, 14]. Briefly, the patients diagnosed as CAD [International Classification of Diseases -10 codes I20-I25] according to World Health Organization 1999/2000 guideline [15–16] were recruited from General Hospital of Guangzhou Military Command of People’s Liberation Army in China between December 2008 and May 2011. The healthy volunteers were enrolled from local communities in Guangzhou during the same period. Participants were excluded if they had type 2 diabetes, parkinson, cirrhosis, chronic kidney diseases, or had been living in Guangdong for less than five years. We initially recruited 1007 CAD patients and 889 healthy controls. After excluding individuals with missing data on dietary intakes (n = 239 of cases and n = 25 of controls), extreme energy intakes (>4000 kcal/d and <800 kcal/d for men, or >3500 kcal/d and <500kcal/d for women, n = 16 of cases and n = 3 of controls) [17], using dietary supplements of n-3 LCPUFAs (n = 15 of cases and n = 2 of controls), or change in dietary habit in the past one year (n = 297 of cases and n = 21 of controls), 440 cases and 838 controls were remained in analysis. No statistical significances were observed between the included and excluded participants in terms of age, sex, BMI, smoking, BP, lipid profiles and variants of *FADS1* rs174547 (**S1 Table**). The study was approved by the ethics committee of Sun Yat-Sen University, and a written informed consent was obtained from all participants.

### Risk factor assessments

Anthropometric indexes including height, weight and blood pressure were measured according to the standardized protocols by trained interns at enrollment. Body mass index was calculated by dividing the weight in kilograms by the square of height in meters. Socio-demographic characteristics (age, residence, education, and marital status), lifestyle habits (smoking, alcohol use and physical activity) as well as medical and family history of diseases were investigated via a structured questionnaire. History of major diseases was collected from an electronic record system. Overnight fasting blood samples were collected and serum lipids were analyzed by an automatic biochemical analyzer in a certified clinical laboratory.

### Dietary intake assessment

Dietary intakes were assessed with a validated quantitative food frequency questionnaire by trained students via a face-to-face interview [18]. The questionnaire consisted of 81 food items with additional items on dietary changes and use of dietary supplements in the past year. The participants were asked to recall the frequency (times per day, week, month, or year) and amounts of food they consumed during the previous year.

Because sea-foods are the major food sources contributing to dietary n-3 LCPUFA (EPA and DHA), the intakes of LCPUFA were assessed by inclusion of fish, molluscs and crustaceans. Fish intakes were estimated based on four sub-groups: freshwater fish (i.e., common carp, grass carp, silver carp, bighead carp, bass, catfish, mandarin fish, eel, yellow eel etc.), marine fish (i.e., salmon, cod, pomfret, tuna, sardine, mackerel pike, Spanish mackerel, red snapper, marlin, grouper etc.), canned fish and salted fish. Molluscs mainly include squid, cuttle fish, octopus, scallop, shellfish, clam, oyster. Crustaceans intakes were estimated from crab and shrimp data(including lobster and little shrimp).

The daily intakes of nutrients and energy were calculated according to the Chinese Food Composition Table [19]. To verify whether the data from food frequency questionnaire is reliable, we detected the erythrocyte membrane EPA and DHA levels in 93 individuals using gas chromatography, and obtained the corresponding diet intake information of EPA and DHA of those 93 individuals from food frequency questionnaire. We applied Spearman correlation analysis to test the relationship between dietary intakes and erythrocyte membrane EPA and DHA levels. The correlation coefficients were 0.371 (*P*<0.001) for EPA and 0.310 (*P*<0.001) for DHA.

### Genotyping

The genotyping of the *FADS1* rs174547 (*T/C*) was performed as previously described [5]. Briefly, genomic DNA was isolated from peripheral blood lymphocytes using the TIANamp Blood DNA Kit (Tiangen, Beijing, China). The rs174547 SNPs were then genotyped using Illumina Golden Gate Genotyping Bead Chips (Illumina, San Diego, CA, USA).

The *FADS1* rs174547 was selected in the present study because: 1) It was the only reported SNP associated with the prevalence of CAD in Chinese population [5]. 2) Epidemiological data suggested that it could interact with dietary n-3 PUFAs to affect LDL-cholesterol concentrations [11]. 3) It was a dominant functional variation in the *FADS* gene cluster that could influence the activity of desaturase [20].

### Statistical analysis

Hardy-Weinberg equilibrium was evaluated for rs174547 in both control group and CAD group using a chi-square test. Genotype of rs174547 is consistent with Hardy-Weinberg equilibrium in control group but not in CAD group. The amounts of dietary n-3 LCPUFA intakes were normalized by Ln-transformation. The general characteristics of participants were compared between cases and controls in terms of age, sex, BMI, socio-demographic factors as well as conventional CAD risk factors. Dietary n-3 LCPUFA intakes were classified into low and high groups according to median of EPA or DHA intakes in control males and females. The cut-points for EPA were 0.52 g/d for males, 0.40 g/d for females, and for DHA were 1.10 g/d for males and 0.88 g/d for females. A dominant genetic model was employed to determine the association between genotypes, dietary n-3 LCPUFA intakes and CAD. Odd ratio (OR) and 95% CIs of *FADS1* rs174547 were estimated using logistic regression models by adjusting for age, gender, body mass index, smoking, education levels, energy intakes, total cholesterol, triglyceride, diastolic blood pressure (DBP), and the history of using aspirin and statins. The interactions between dichotomized n-3 LCPUFA intakes and genotypes on CAD were evaluated through the logistic regression models added with an “interaction term”(variable A× variable B), in which the variables A, B represent SNP rs174547 and the intakes of EPA or DHA, respectively, In a multiple logistic regression, the interaction effects of the two variables A and B are usually indicated by their product A×B which called “interaction term”. So *P* value of the additional interaction term derived from the logistic model represent the *P*-interaction between rs174547 and n-3 LCPUFA on CAD.Statistical analysis was performed with SPSS software (version 16.0, SPSS Inc.). All reported *P* values were 2-tailed and *P*<0.05 was considered statistically significant.

## Results

### Population characteristics

The characteristics of cases and controls are shown in **Table 1**. CAD patients were more likely to be older, have higher body mass index, triglycerides and systolic blood pressure, more smoking and alcohol usage, whereas lower HDL-cholesterol. Since most of the patients took medicines to regulate their hypertension and lipids, they had lower total cholesterol, LDL-cholesterol and DBP compared to the controls.

**Table 1:** General characteristics of the case-control study population.

	Control (n = 838)	Case (n = 440)	*P* value
Age (y)	59.1 ± 5.2	62.9 ± 11.2	<0.001
Male (n, %)	531, 63.1	303, 68.9	0.056
Body mass index (kg/m^2^)	23.25 ± 3.18	24.05 ± 3.62	<0.001
Smoking (%)	34.0	39.8	0.043
Marriage (%)			0.34
Single	1.3	0.5	
Married	90.2	90.7	
Separated	8.4	8.8	
Education			<0.001
< High school	33.9	59.7	
High school	40.4	22.4	
> High school	25.7	17.0	
Lipids (mmol/L)			
Total cholesterol	5.14 ± 0.95	4.65 ± 1.05	<0.001
Triglycerides	1.50 ± 1.10	1.72 ± 1.03	<0.001
LDL-cholesterol	3.47 ± 0.83	2.95 ± 0.99	<0.001
HDL-cholesterol	1.32 ± 0.33	1.09 ± 0.31	<0.001
Blood pressure (mmHg)			
Systolic blood pressure	122.92 ± 16.69	131.78 ± 21.29	<0.001
Diastolic blood pressure	77.77 ± 9.95	75.46 ± 12.48	0.001
Diseases (%)			
Hypertension	0.0	57.2	<0.001
Hyperlipidemia	0.0	35.2	<0.001
Medication treatment (%)			
Antihypertensive drugs	0.0	48.4	<0.001
Aspirin	0.0	20.4	<0.001
Statins	0.0	16.1	<0.001
Dietary intakes			
Energy (kcal/d)	1802 ± 477	1758 ± 616	0.16
Total fatty acid (g/d)	59.59 ± 21.90	57.57 ± 28.41	0.16
n-3 LCPUFA intakes (g/d)			
EPA intakes	0.47 (0.23, 0.90)	0.23 (0.08, 0.58)	<0.001
DHA intakes	1.02 (0.51, 1.86)	0.58 (0.21, 1.40)	0.003

Abbreviations: HDL, high density lipoprotein; LDL, low density lipoprotein; LCPUFA, long-chain polyunsaturated fatty acid; EPA, eicosapentaenoic acid; DHA, docosahexaenoic acid.

### Association of *FADS1* with the risk of CAD

We conducted a genotype analysis of the rs174547 polymorphism in the study. As shown in **Table 2**, after adjustment for age, sex, body mass index, smoking, total cholesterol, triglyceride, diastolic blood pressure, education, history of using aspirin and statins, the OR for CAD was (OR = 1.36, 95% CIs 1.03–1.80) for *TC/TT* subjects compared to those homozygous *C* allele in the dominant model. Additionally, we test the multicollinearity of the explanatory variables included in our multiple logistic regression models. Data showed that all the explanatory variables are independent to each other and no multicollinearity was observed.

**Table 2:** Association between the genetic polymorphism in *FADS1* rs174547 with CAD^a^.

Risk allele	OR (95% CIs)		
	*CC* (n = 598)	*TC/TT* (n = 680)	*P* value
*T*	1.00	1.36 (1.03–1.80)	0.029

^a^ Adjusted for age, sex, body mass index, smoking, total cholesterol, triglyceride, diastolic blood pressure and education. the history of using statins.

Abbreviations: CAD, coronary artery disease.

The results of the additive genetic model were similar to those in the dominant model. The minor *T* allele was associated with higher CAD risk, (OR = 1.00 for *CC* vs OR = 1.23, 95% CIs 0.92–1.66 for *TC* vs OR = 1.88, 95% CIs 1.22–2.89 for *TT*) after adjusting for all the potential confounders in model 3 (**S2 Table**). No multicollinearity was revealed in the additive genetic model either.

### Nutrigenetic interaction of n-3 LCPUFA with *FADS1* on CAD

We further examined whether the association of FADS1 with CAD risk was modulated by dietary n-3 LCPUFA intakes. Due to increased CAD risk in the variant *T* allele carrier, we used *CC* homozygote with high dietary n-3 LCPUFA group as the reference group. After adjustment for age, sex, body mass index, smoking, total cholesterol, triglyceride, diastolic blood pressure, education, history of using aspirin and statins, the interaction of dietary EPA intakes with rs174547 on CAD was statistically significant (*P*-interaction = 0.028) in the dominant genetic model (**Table 3**), In the low EPA intake group, subjects with *T* allele were at higher risk of CAD than those with homozygous of *C* allele (OR = 3.04, 95%CI 1.94–4.76 versus OR = 1.87, 95%CI 1.17–2.98). However, this association was not observed in subjects with high EPA intake. Gene-dietary interaction analyses using an additive genetic model also yielded consistent results (**S3 Table**). Test of multicollinearity was applied in both dominant and additive genetic model and no multicollinearity were found.

**Table 3:** Nutrigenetic interaction of EPA and DHA with *FADS1* rs174547 on risk of CAD under a dominant genetic model^a^.

	Genotype groups				
	*CC*		*TC/TT*		*P*-interaction
	n	OR (95% CIs)	n	OR (95% CIs)	
EPA					0.028
High	265	1.00	280	0.81 (0.49–1.34)	
Low	333	1.87 (1.17–2.98)	400	3.04 (1.94–4.76)	
DHA					0.020
High	275	1.00	283	0.82 (0.51–1.32)	
Low	323	1.52 (0.95–2.42)	397	2.56 (1.64–3.98)	

^a^ Adjusted for age, sex, body mass index, smoking, total cholesterol, triglyceride, diastolic blood pressure, education and energy intakes, the history of using statins.

Abbreviations:; HAenergy intakes. EPA, eicosapentaenoic acid; CAD, coronary artery disease; DHA, docosahexaenoic acid.

Similar findings were observed in dietary DHA intakes (**Table 3**). There was a significant interaction (*P*-interaction = 0.020) between DHA intake and genetypes on CAD. In this model, subjects with *TC/TT* exhibited higher risk of CAD (OR = 2.56, 95%CIs 1.64–3.98) than those with *CC* genotype (OR = 1.52, 95%CIs 0.95–2.42) under a low DHA intake, but not in high DHA intake group (OR = 0.82, 95% CIs 0.51–1.32 versus OR = 1.00). Consistent results were also observed under an additive genetic model (**S3 Table)**.

## Discussion

In the current study, we observed that carrier of rs174547 minor *T* allele was a risk factor of CAD. The associations were modulated by dietary EPA and DHA intakes with signficant increased CAD in that lower intake of EPA or DHA.

Our findings were consistent with previous reports [5,18]. An earlier case-control study also reported that variations in the haplotypes of *FADS1-FADS2-FADS3* gene cluster were associated with greater risk of CAD [21]. However, inconsistent associations were observed between genetic variation of *FADS1* rs174547 and blood lipid/lipoprotein, the classical risk factors of CAD. In the Korean population, the minor allele of *FADS1* rs174547 was associated with decreased HDL-cholesterol and increased triglyceride concentrations, whereas decreased LDL-cholesterol concentrations [22]. Nakayama *et al*. [3] reported similar results in two genetically similar Asian populations. One was in a Japanese population, in which the minor allele of rs174547 was associated with lower HDL-cholesterol and higher triglyceride concentrations. However, in Mongolian population, the minor allele of rs174547 was associated with decreased LDL-cholesterol concentrations.

The discrepancy could be due to different population characteristics, covariates adjusted, or the differences in medical treatment on lipids and BP, along with other potential CAD risk factors (i.e., dietary fatty acid intake) that were not fully controlled. So we choose CAD as an end-point outcome in our study instead of risk factors such as blood lipids.

In this study, we found that n-3 LCPUFA could interact with *FADS1* rs174547 *T* allele to affect the risk of CAD. Our data demonstrated that the rs174547 minor *T* risk allele carriers had higher risk of CAD with low intake of EPA or DHA. The result was consistent with previous studies, which found that n-3 LCPUFA (or alpha-linolenic acid of n-3 PUFAs) intakes modified the association between genetic polymorphism of *FADS1* and blood lipids/lipoprotein concentrations [23]. For example, in a study conducted in Sweden, significant interaction between rs174547 and long chain-3 PUFA intakes on LDL was found. Different n-3 PUFA intakes were converted into the percentage contributed by the specific n-3 PUFA to total energy intakes (E%), with the points of 0.14E% and 0.28E% dividing the n-3 PUFA intakes of 4635 individuals into three different levels, the result of which showed that the C-allele was only associated with lower LDL among individuals in the lowest tertile of long-chain n-3 PUFA intakes [11]. Significant associations between rs174546 genotypes and concentrations of total and non-HDL-cholesterol were observed in the group with high intake of n−3 PUFAs but not in the low-intake group in a Netherlands population [12].In another study conducted in European adolescents, rs174546 minor allele was also found associated with lower total cholesterol concentrations and non-HDL-cholesterol concentrations in the high-α-linolenic acid (ALA)-intake group but not in the low-ALA-intake group [13]

The underlying mechanism of the interaction among FADS1 genetic polymorphism, n-3 LCPUFA and CAD was still unclear. Besides dislipidemia, inflammation is also a crucial factors in the progression of CAD [24]. In vivo, the minor variation of *FADS1* may increase the levels of AA(derived from n-6 fatty acid LA), precursor of important proinflammatory molecules such as eicosanoids, which might induce a proinflammatory response linking to the progression of CAD [25], while EPA derived from n-3 fatty acid ALA presents anti- inflammatory effect. From a quantitative point of view, LA was almost100 times as abundant as ALA, and thus AA was almost 30 times as abundant as EPA. Martinelli *et al*. [21] suggested that prevalence of n-6 FAs may account for the pro-inflammatory effect observed in CAD patients, and the detrimental effects of increased desaturase activity may be exaggerated by diets abundant in n-6 and relatively deficient in n-3 FAs, so n-3 PUFA supplementation can enhance cell EPA whereas reduce AA level, thus protecting against increased desaturase activity in patients with unfavorable FADS alleles. n-3 PUFA supplementation would be one of the therapeutic strategies in patients with a genetically determined increase in desaturase activity. Therefore, high dietary intakes of n-3 LCPUFA, which have been considered as a preventive factor against CAD for its anti-inflammation effect [26], may negate the inflammation response of AA. However, the molecular mechanism still needs to be elucidated in future studies.

To our knowledge, it’s the first report observed that direct effect of n-3 PUFA× rs174547 interaction on CAD. However, there were a number of limitations which should be addressed. Firstly, the study was a case-control design and can not address whether the association we found is truly casual or of pathologic significance. Secondly, we had also included the CAD patients who are not newly diagnosed, but excluded patients (n = 297) who changed their dietary habits in recent one year. Stratification analysis using patients who are newly or not newly diagnosed indicated similar findings (data not shown), which suggested a limited reversal association. Lastly, the dietary intakes were obtained from a validated food frequency questionnaire which required the participants to recall the average food consumption in the last year, which may lead to recall bias. However, the bias introduced from non-differential misclassification could only lead to an underestimation, rather than an overestimation [27]. Moreover, we validated the reliability of dietary n-3 LCPUFAs intakes from questionnaire by comparing with their corresponding erythrocyte membrane FA composition, the latter considered as objective markers which can represent dietary FA intakes to some extent. We found that they were well correlated.

In conclusion, we found that high dietary n-3 LCPUFA intakes could negate the unfavorable effect of genetic variation in *FADS1* on CAD in middle-aged and elderly population. Future comprehensive designed longitudinal studies are needed to further confirm our findings and explore the potential mechnism.

## Supporting Information

S1 Table Characteristics of excluded and included participants. (DOC)

S2 Table Association of *FADS1* rs174547 with CAD under an additive model. (DOC)

S3 Table Nutrigenetic interaction of dietary EPA and DHA intakes with *FADS1* rs174547 on risk of CAD under an additive model. (DOC)
