# Supplementary material for: Dietary n-3 Polyunsaturated Fatty Acid Intakes Modify the Effect of Genetic Variation in Fatty Acid Desaturase 1 on Coronary Artery Disease
Source: PLoS One. 2015 Apr 7;10(4):e0121255. doi: 10.1371/journal.pone.0121255 (PMC4388373; doi:10.1371/journal.pone.0121255)
Supplement: S1 Table — (DOC) [file pone.0121255.s001.doc]

**S1 Table. Characteristics of excluded and included participants**

|  | controls | | *P*  value | cases | | *P*  value |
| --- | --- | --- | --- | --- | --- | --- |
|  | Included  n = 838 | Excluded  n= 51 |  | Included  n = 440 | Excluded  n= 567 |  |
| Age (y) | 59.1 ± 5.2 | 59.9 ± 3.6 | 0.058 | 62.9 ± 11.2 | 63.6 ± 10.9 | 0.278 |
| Male (n, %) | 531, 63.1 | 30, 58.8 | 0.487 | 303, 68.9 | 378, 66.7 | 0.577 |
| Body mass index (kg/m^2^) | 23.25 ± 3.18 | 22.91 ± 3.14 | 0.245 | 24.05 ± 3.62 | 23.67 ± 3.21 | 0.090 |
| Smoking (%) | 34.0 | 29.5 | 0.477 | 39.8 | 41.8 | 0.292 |
| Lipids/lipoprotein (mmol/L) | | | | | | |
| Total cholesterol | 5.14 ± 0.95 | 5.16 ± 0.96 | 0.793 | 4.65 ± 1.05 | 4.70 ± 1.10 | 0.433 |
| Triglyceride | 1.50 ± 1.10 | 1.37 ± 0.62 | 0.067 | 1.73 ± 1.03 | 1.86 ± 1.32 | 0.086 |
| LDL-cholesterol | 3.47 ± 0.83 | 3.47 ± 0.87 | 0.997 | 2.95 ± 0.99 | 2.98 ± 0.97 | 0.677 |
| HDL-cholesterol | 1.32 ± 0.33 | 1.32 ± 0.28 | 0.885 | 1.09 ± 0.31 | 1.09 ± 0.30 | 0.896 |
| Blood pressure (mm Hg) |  |  |  |  |  |  |
| Systolic blood pressure | 122.92 ± 16.69 | 122.13 ± 13.30 | 0.535 | 131.16 ± 21.29 | 132.16 ± 20.88 | 0.782 |
| Diastolic blood pressure | 77.77 ± 9.95 | 76.06 ± 8.26 | 0.057 | 75.46 ± 12.48 | 76.89 ± 12.48 | 0.071 |
| *FADS1* rs174547 (%) |  |  | 0.170 |  |  | 0.209 |
| *CC* | 48.3 | 41.7 |  | 43.9 | 38.1 |  |
| *TC* | 40.0 | 44.9 |  | 40.0 | 46.2 |  |
| *TT* | 11.7 | 13.4 |  | 16.1 | 15.7 |  |

LDL, low density lipoprotein; HDL, high density lipoprotein.
