# Supplementary material for: Dietary n-3 Polyunsaturated Fatty Acid Intakes Modify the Effect of Genetic Variation in Fatty Acid Desaturase 1 on Coronary Artery Disease
Source: PLoS One. 2015 Apr 7;10(4):e0121255. doi: 10.1371/journal.pone.0121255 (PMC4388373; doi:10.1371/journal.pone.0121255)
Supplement: S2 Table — (DOC) [file pone.0121255.s002.doc]

**S2 Table. Association of *FADS1* rs174547 with CAD under an additive model**

| Risk  allele | OR (95% CIs) | | | | |
| --- | --- | --- | --- | --- | --- |
|  | *CC*  n = 598 | | *TC*  n = 510 | *TT*  n = 169 | *P*- trend |
| *T* | 1.00 | 1.23 (0.92-1.66) | | 1.88 (1.22-2.89) | 0.014 |

Adjusted for age, gender, BMI, smoking, education.total cholesterol, triglyceride,

diastolic blood pressure，history of using aspirin，statins.

CAD, coronary artery diease.
