# Supplementary material for: Dietary n-3 Polyunsaturated Fatty Acid Intakes Modify the Effect of Genetic Variation in Fatty Acid Desaturase 1 on Coronary Artery Disease
Source: PLoS One. 2015 Apr 7;10(4):e0121255. doi: 10.1371/journal.pone.0121255 (PMC4388373; doi:10.1371/journal.pone.0121255)
Supplement: S3 Table — (DOC) [file pone.0121255.s003.doc]

**S3 Table. Nutrigenetic interaction of dietary EPA and DHA intakes with *FADS1* rs174547 on risk of CAD under an additive model**

|  | Genotype group | | | | | | *P* -interaction |
| --- | --- | --- | --- | --- | --- | --- | --- |
|  | *CC* | | *TC* | | *TT* | |
|  | n | OR(95% CIs) | n | OR(95% CIs) | n | OR(95% CIs) |
| EPA |  |  |  |  |  |  | 0.048 |
| High | 265 | 1.00 | 211 | 0.71 (0.41-1.23) | 69 | 1.18 (0.56-2.49) |  |
| Low | 333 | 1.85 (1.16-2.95) | 300 | 2.69 (1.68-4.31) | 100 | 4.38 (2.37-8.12) |  |
| DHA |  |  |  |  |  |  | 0.036 |
| High | 275 | 1.00 | 217 | 0.72 (0.43-1.23) | 66 | 1.17 (0.56-2.45) |  |
| Low | 323 | 1.50 (0.94-2.39) | 294 | 2.23 (1.42-3.62) | 103 | 3.58 (1.95-6.57) |  |

Adjusted for age, gender, BMI, smoking, education, energy intakes, TC, TG and DBP, history of using aspirin and statins.

EPA, eicosapentaenoic acid; DHA, docosahexaenoic acid; CAD, coronary artery disease.
